# Correction: Robertson et al. Al^3+^ Modification of Graphene Oxide Membranes: Effect of Al Source. *Membranes* 2022, *12*, 1237

**DOI:** 10.3390/membranes15010028

**Published:** 2025-01-14

**Authors:** Ellen J. Robertson, Yijing Y. Stehle, Xiaoyu Hu, Luke Kilby, Katelyn Olsson, Minh Nguyen, Rebecca Cortez

**Affiliations:** 1 Chemistry Department, Union College, Schenectady, NY 12308, USA; 2 Department of Mechanical Engineering, Union College, Schenectady, NY 12308, USA

The authors wish to make a correction to the published paper [1]. In the original publication, there was a mistake in the interlayer distance calculation from the XRD spectrum data in Section 3.2, 15th paragraph (the last paragraph of the section). The error occurred because the unit of the diffraction angle was processed as radians instead of degrees in Excel using the SIN function.

In Section 3.2, last paragraph, the sentence

“Inserting Al^3+^ into the gallery spaces between the GO sheet basal planes increased the d-spacing from 4.00 nm for the unmodified GO membrane to 4.28 nm for the AGO (Al_2_O_3_) membrane, to 4.37 nm for the AGO (AlCl_3_) membrane, and 4.16 nm for the AGO (Al foil) membrane (Figure S4).”

should be corrected to the following:

“Inserting Al^3+^ into the gallery spaces between the GO sheet basal planes increased the d-spacing from 0.77 nm for the unmodified GO membrane to 0.85 nm for the AGO (Al_2_O_3_) membrane, to 0.87 nm for the AGO (AlCl_3_) membrane, and 0.83 nm for the AGO (Al foil) membrane (Figure S4).”

The same correction applies to Figure S4, where the average d-spacing values for the unmodified and modified GO, AGO membranes determined from the XRD spectra should be updated accordingly.

**Figure S4:**
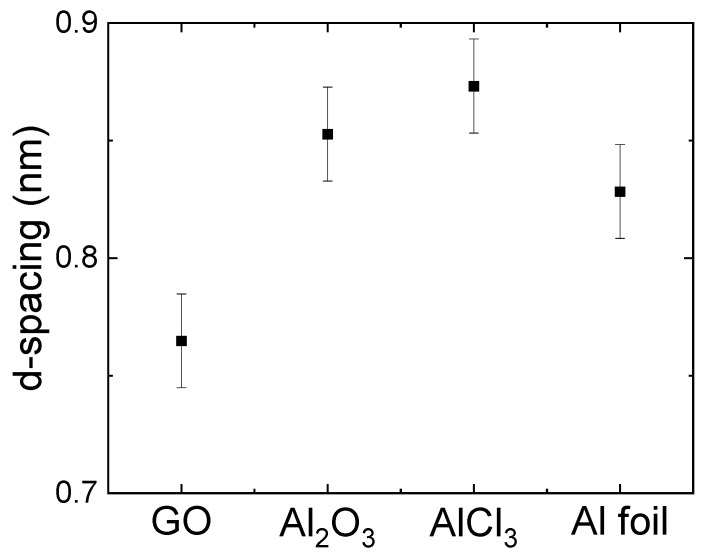
Average d-spacing values for the unmodified GO, AGO (Al_2_O_3_), AGO (AlCl_3_), and AGO (Al foil) membranes determined from the XRD spectra.

The authors state that the scientific conclusions are unaffected. This correction was approved by the Academic Editor. The original publication has also been updated.
