# Supplementary material for: Al3+ Modification of Graphene Oxide Membranes: Effect of Al Source
Source: Membranes (Basel). 2022 Dec 7;12(12):1237. doi: 10.3390/membranes12121237 (PMC9788489; doi:10.3390/membranes12121237)
Supplement: Supplementary file 1 [file membranes-12-01237-s001.zip › membranes-2024694-supplementary.pdf]

Al^{3+} Modification of Graphene Oxide Membranes: Effect of Al Source

Ellen J. Robertson[1,*], Yijing Y. Stehle[2,*], Xiaoyu Hu[2,], Luke Kilby[2], Katelyn Olsson[2], Minh Nguyen[2], and Rebecca Cortez[2]

[1] Chemistry Department, Union College, Schenectady, NY 12308, USA; roberte2@union.edu
[2] Department of Mechanical Engineering, Union College, Schenectady, NY 12308, USA; stehley@union.edu
***** Correspondence: roberte2@union.edu; stehley@union.edu

Table of Contents

1. AFM images of GO and AGO membranes.................................... 2
2. UV-visible spectroscopy of GO and AGO membranes 3
3. Elemental mapping of GO and AGO membranes 4
4. Membrane d-spacing values 5
5. Membrane flux and permeability.. 6
6. References 7

AFM images of GO and AGO membranes

Atomic force microscope (AFM) images of the GO and AGO membranes were analyzed to characterize the surface roughness of each membrane. Representative AFM images are shown in Figure S1.

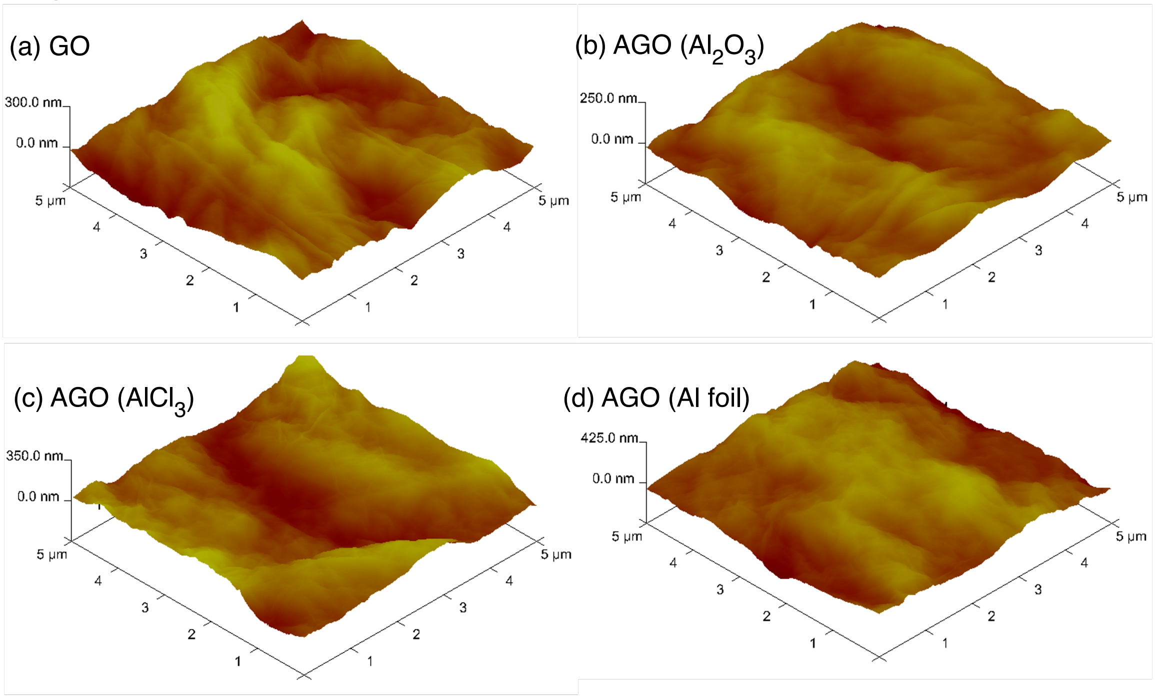

Figure S1. AFM images of the GO (a), AGO (Al_2O_2) (b), AGO ($AlCl_3$) (c), and AGO (Al foil) (d) membranes.

UV-visible spectroscopy of GO and AGO membranes

The UV-visible spectra (Fig. S2) were obtained for the unmodified GO and all AGO membranes. The spectra were very similar to each other, which suggests that the introduction of Al^{3+} to the membrane from either Al_2O_3, $AlCl_3$, or Al foil did not significantly result in the partial reduction of the GO membrane.

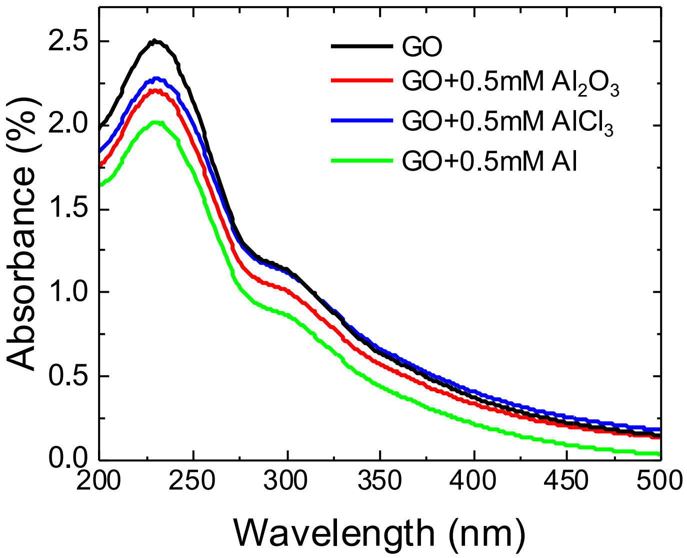

Figure S2: UV-visible spectra of the unmodified GO membrane (black trace), the AGO (Al_2O_3) membrane (red trace), AGO ($AlCl_3$) membrane (blue trace) and AGO (Al foil) membrane (green trace).

Elemental mapping of GO and AGO membranes

SEM images of the unmodified GO and each AGO membrane were collected along with corresponding elemental maps for carbon, oxygen, and aluminum obtained using EDS (Fig. S3). The mapping shows an even lateral distribution of carbon and oxygen throughout each membrane, and an even lateral distribution of aluminum throughout AGO (Al foil) and AGO ($AlCl_3$) membranes.

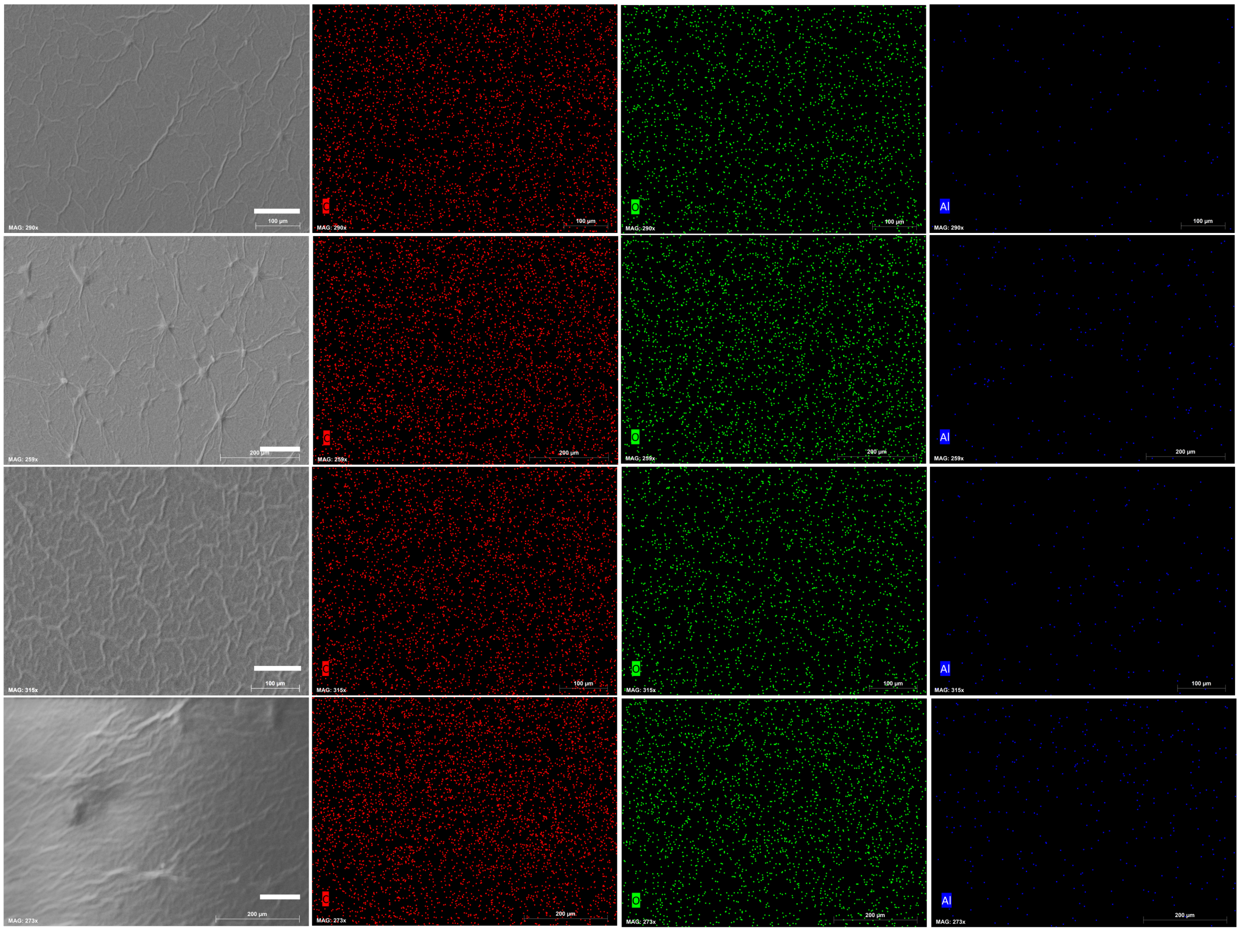

Figure S3: SEM images and corresponding carbon (red), oxygen (green), and aluminum (blue) maps for the unmodified GO, AGO (Al_2O_3), AGO ($AlCl_3$), and AGO (Al foil) membranes.

Membrane d-spacing values

X-ray photoelectron spectra were used to determine the interlayer spacing, or d-spacing, for each membrane (Fig. S4). The d-spacing of the unmodified GO and the AGO membranes were calculated using Bragg's law (Equation S1), where $\lambda = 0.15418$ nm is the wavelength of the X-ray beam, d is the membrane's interlayer spacing, and 2θ is the diffraction angle.

$$\lambda = 2dsin\theta \qquad \text{(Equation S1)}$$

Here, the interlayer spacing increased for all AGO membranes relative to that of the unmodified GO membrane.

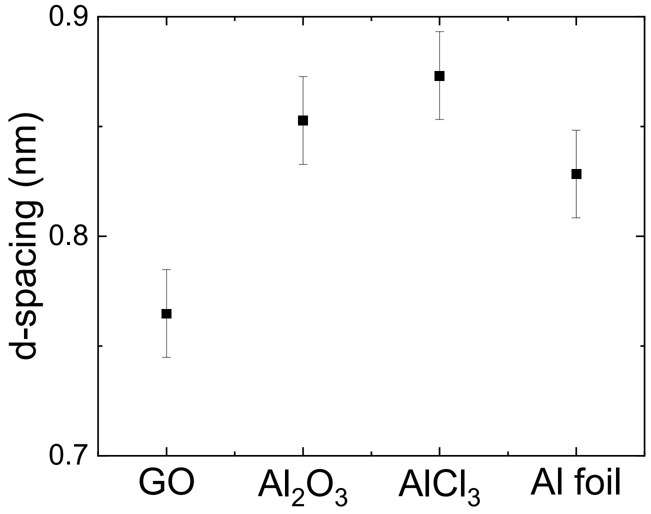

Figure S4. Average d-spacing values for the unmodified GO, AGO (Al_2O_3), AGO ($AlCl_3$), and AGO (Al foil) membranes determined from the XRD spectra.

Membrane flux and permeability

The permeation performances of GO and AGO membranes for water vapor were measured using a simple evaporation device [1]. The weight difference (M, g) of the solvent was measured by periodically monitoring the mass of the vial for up to seven days and used in the permeation rate calculation (Equation S2). The flux rate (J, g/(m^2hr)) of water vapor across membranes was determined from the slope of the curve between the amount of solute molecule permeated per unit membrane area (n/A) and the permeation time (t).

$$J = \frac{M}{At} \qquad \text{(Equation S2)}$$

The flux data were used to calculate the diffusion coefficient, D, according to Equation S3, where ΔC is the concentration of the vapor on the two sides of the membrane and Δx is the membrane thickness. Here, we equate the diffusion coefficient to the membrane permeability.

$$J = \frac{-D\Delta C}{\Delta x} \qquad \text{(Equation S3)}$$

Figure S5 shows the flux (a) and permeability (b) results for the unmodified GO membrane and the AGO membranes as a function of temperature.

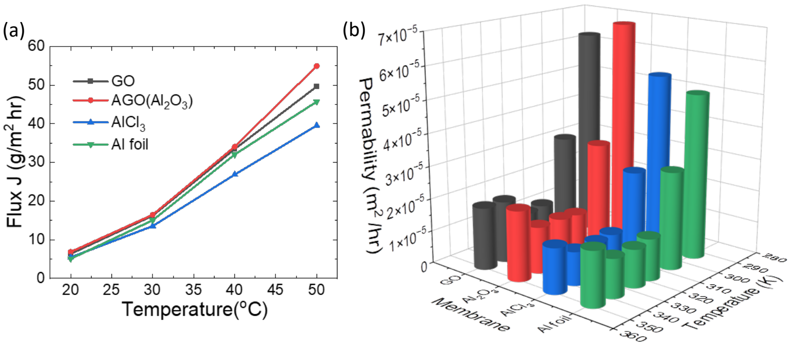

Figure S5. (a) Measurements of the flux of water through the GO, AGO (Al_2O_3), AGO ($AlCl_3$), and AGO (Al foil) membranes as a function of temperature and (b) the corresponding water permeability values. Each recorded permeability value is the mean of at least 5 experiments at the same temperature.

Compared to the unmodified GO membrane, the AGO (Al_2O_3) membrane showed an increase in flux and permeability values, while the AGO ($AlCl_3$) and AGO (Al foil) membranes showed a decrease in flux and permeability values at all temperatures measured. We attribute the increase in the water permeability for the AGO (Al_2O_3) membrane in part to the insertion of unreacted alumina particles into the membrane structure, which increased the nanochannel sizes relative to the unmodified GO membrane. We attribute the decrease in water permeability for the AGO ($AlCl_3$) and AGO (Al foil) membranes in part to Al^{3+} crosslinking to the carboxylate groups on the

edges of the GO sheets, as noted in our previous study [1]. Along with the extent of Al^{3+} crosslinking in the GO membrane, there are other factors that can affect the membrane permeability to water. Specifically, both membrane thickness (Δx) and differences in the membrane surface and interior structures can affect the extent that water vapor passes through both unmodified and modified GO membranes [1]. In our study, the membrane thickness could be measured to a precision of only 2 mm. Moreover, based on the surface tension results reported in this work, it is very likely that the surface and interior structures of all membranes studied were very different from each other. Thus, it is hard to say with certainty if differences in membrane permeability were entirely due to Al^{3+} modification, or if the membrane thickness and overall structure also affected the permeability.

References

[1] Stehle, Y.Y.; Robertson, E.J.; Cortez, R.; Vlassiouk, I.V.; Bucinell, R.B.; Olsson, K.; Kilby, L. Using Al^{3+} to Tailor Graphene Oxide Nanochannels: Impact on Membrane Stability and Permeability. *Membranes* **2022**, *12*, 871.
